# DNA binding strength increases the processivity and activity of a Y-Family DNA polymerase

**DOI:** 10.1038/s41598-017-02578-3

**Published:** 2017-07-06

**Authors:** Jing Wu, Alexandra de Paz, Bradley M. Zamft, Adam H. Marblestone, Edward S. Boyden, Konrad P. Kording, Keith E. J. Tyo

**Affiliations:** 1 Department of Chemical and Biological Engineering, Northwestern University, Evanston, Illinois United States of America; 2 School of Pharmaceutical Science, Jiangnan University, Wuxi, Jiangsu Province China; 3 Interdisciplinary Biological Sciences Program, Northwestern University, Evanston, Illinois United States of America; 4 Department of Genetics, Harvard Medical School, Boston, Massachusetts United States of America; 5 Biophysics Program, Harvard University, Boston, Massachusetts United States of America; 6 Wyss Institute, Harvard University, Boston, Massachusetts United States of America; 7 Media Lab, Massachusetts Institute of Technology, Cambridge, Massachusetts United States of America; 8 Department of Biological Engineering, Massachusetts Institute of Technology, Cambridge, Massachusetts United States of America; 9 McGovern Institute, Massachusetts Institute of Technology, Cambridge, Massachusetts United States of America; 10 Department of Physiology, Northwestern University, Chicago, Illinois United States of America; 11 Department of Applied Mathematics, Northwestern University, Chicago, Illinois United States of America

## Abstract

DNA polymerase (pol) processivity, i.e., the bases a polymerase extends before falling off the DNA, and activity are important for copying difficult DNA sequences, including simple repeats. Y-family pols would be appealing for copying difficult DNA and incorporating non-natural dNTPs, due to their low fidelity and loose active site, but are limited by poor processivity and activity. In this study, the binding between Dbh and DNA was investigated to better understand how to rationally design enhanced processivity in a Y-family pol. Guided by structural simulation, a fused pol Sdbh with non-specific dsDNA binding protein Sso7d in the N-terminus was designed. This modification increased *in vitro* processivity 4-fold as compared to the wild-type Dbh. Additionally, bioinformatics was used to identify amino acid mutations that would increase stabilization of Dbh bound to DNA. The variant SdbhM76I further improved the processivity of Dbh by 10 fold. The variant SdbhKSKIP241–245RVRKS showed higher activity than Dbh on the incorporation of dCTP (correct) and dATP (incorrect) opposite the G (normal) or 8-oxoG(damaged) template base. These results demonstrate the capability to rationally design increases in pol processivity and catalytic efficiency through computational DNA binding predictions and the addition of non-specific DNA binding domains.

## Introduction

As we study and engineer organisms, there is a demand for more sophisticated strategies for cloning, mutagenizing, labeling, detecting, and amplifying DNA. To meet this demand, there is an increasing need to modulate a range of DNA polymerase (pol) properties, beyond what exists in nature^1, 2^. Processivity, the average number of bases a pol will extend before falling off a template, and catalytic properties, such as V_max_ and K_m_, are useful properties to modulate. High processivity is important for efficiently copying simple repetitious sequences^3, 4^, and high incorporation rates are important to be applicable for biotechnology applications^5^.

Y-family pols are a superfamily of evolutionarily related proteins that exist in cells to bypass DNA damage caused by both radiation and chemicals^6^. Dpo4 from *Sulfolobus solfataricus*, is the most characterized Y-family polymerase. However, Dbh (DinB homologue) from *S*. *acidocaldarius*, as a close relative of Dpo4, is particularly interesting for pol-based biotechnology applications. Dbh has a relatively high nucleotide misincorporation ratio (the enzyme exhibits misinsertion fidelities in the range of 8 × 10^−3^ to 3 × 10^−4^ on undamaged DNA templates, as determined by steady-state kinetic analysis) that could be useful for mutagenesis at mesophilic conditions^7–9^. However, Y-family pols typically have low processivity (only able to polymerize a limited number of nucleotides during a single association-dissociation cycle) and activity/incorporation rate, limiting their usefulness *in vivo* and *in vitro* ^10^. Improving the processivity of Y-family pols, while increasing or maintaining catalytic activity, would enhance the utility of this pol for mutagenesis studies on difficult to replicate DNA.

Y-family pols have important structural differences compared to other pols that contribute to their low fidelity and processivity^11–13^. High processivity is an important attribute of all *replicative* pols, which have a typical overall structure shaped like a right hand, composed of thumb, finger, and palm domains^14^. The catalytic carboxylate-metal ion complex sits in the palm domain while the mobile finger and thumb domains grasp the template and primer to create an active site that aligns the template base and incoming nucleotide^14, 15^. Y-family pols have similar structures to replicative pols, however the fingers and thumb domains are smaller than most other polymerases, resulting in virtually no contact with the major-groove side of the nascent base pair by the fingers, and the thumb making fewer contacts with both the DNA substrate and incoming nucleotide. These structural differences contribute to the decreased processivity and fidelity of Y-family pols compared to other pol families^16^. Furthermore, Y-family pols have an additional fourth domain called the “little finger” (LF) or “polymerase associated domain” (PAD), which helps grip on the DNA and has also been associated with the family’s low fidelity and translesion replication capabilities^17–19^.

Thus, compared to the catalytic process of high-processivity pols, the Y-family pols impose relatively few constraints on the nascent base pair and DNA substrates^13, 17^. This lack of constraints results in simultaneously reduced fidelity and processivity. However, structure may not be the only determinant of processivity. Increased binding affinity of pols to DNA would likely increase processivity, as the pol is less likely to fall off the DNA. In this scenario, the relaxed active site would still readily accommodate misincorporation, but tighter binding might improve processivity.

Several previous studies have shown that altering DNA binding properties, through adding DNA binding domains and introducing mutations, can affect processivity and catalytic activity^20–23^. For example, the dsDNA binding domain, Sso7d (7 kDa) from *S*. *solfataricus*, binds to DNA in a sequence-independent manner. The processivity of both A-family and B-family polymerases has been shown to be enhanced by fusing the polymerases with Sso7d^24^.

Certain mutations are also known to affect processivity and catalytic efficiency of Y-family pols^25^. For example, a non-conserved residue Arg332 within the LF domain has been shown to be responsible for DNA binding and DNA translocation after phosphodiester bond formation past damaged DNA(8-oxoG) in Dpo4^26^. The Ala332 and Glu332 mutants were each 2-fold faster at full-length extension opposite unmodified DNA than WT Dpo4^26^. Likewise, increasing glycine content in the linker region between the thumb and LF domain of Dpo4 was shown to decrease DNA binding affinity by 250-fold compared to the wild-type^27^.

In the present study, we evaluated the effect of pol/DNA binding on processivity and catalytic activity for Dbh by (a) tethering the pol to a non-specific DNA binding domain and (b) introducing point mutations in the pol that increase the binding to DNA. In both cases, we anticipated that increasing DNA binding would increase processivity. We attached Sso7d to the N-terminus of Dbh and introduced mutations that were computationally predicted to strengthen binding free energy (Δ*G*_*binding*_). Both strategies resulted in increased processivity during extension of a single-stranded DNA (ssDNA) template, M13mp18. The addition of Sso7d did not change kinetic properties, and several computationally guided mutations improved k_*cat*_. This is a step toward the broader utility of Dbh in biotechnology.

## Results

### Generation of Sso7d-Dbh fusion protein (Sdbh)

**Figure 1:**
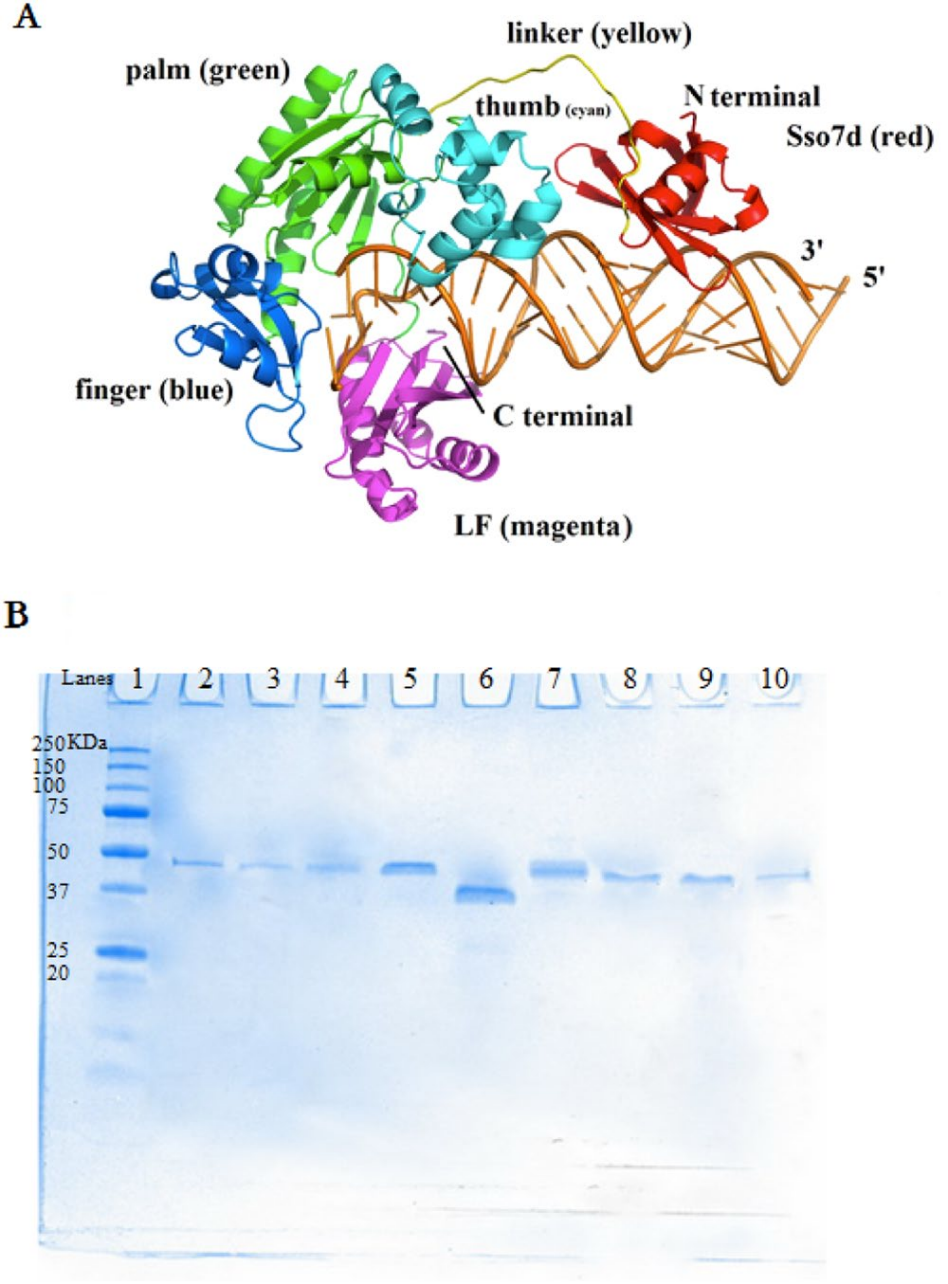
(**A**) A ribbon diagram of Dbh complexed with Sso7d and DNA. The location of the four structural domains in Dbh are color coded as follows: green, palm domain; cyan, thumb domain; blue, finger domain; magenta, little finger (LF) domain; red, Sso7d. Sso7d is located at the N-terminus and is covalently linked to the palm domain. Sso7d binds to DNA by placing a triple-stranded beta-sheet across the DNA minor groove. The amino acid sequence of the flexible linker (yellow) is SS(GGGGS)_3_GM. The DNA substrate is shown in brown. (**B**) SDS-PAGE of the purified Dbh and the variants. Samples were separated on a 10% Mini-PROTEAN TGXTM precast gel (Bio-Rad) and proteins were visualized after staining with Coomassie Brilliant Blue R-250. Lane 1, protein marker (protein molecular weight (kDa) was showed on the left of the lane 1); Lane2-10, SdbhM76I, SdbhKSKIP241–245RVRKS, SdbhL250V, Sdbh, Dbh, SdbhT37F, SdbhA221S, SdbhI62V and SdbhK337R, respectively.

Sso7d was covalently linked to Dbh to investigate the role that Sso7d could play on the processivity of Y-family pols. Because steric constraints between Sso7d and Dbh could prohibit Sso7d from interacting with the DNA template, we used a structure-guided approach to design an appropriate linker. Using the published 3D structures of Dbh and Sso7d, we simulated the binary complexes of Sdbh-DNA. A flexible linker SS(GGGGS)_3_GM was found to tether Sso7d to the palm domain of Dbh while maintaining protein-DNA interactions for both Dbh and Sso7d (Fig. 1A). Sso7d was far away from the active site of Dbh on the spatial structure and was predicted to only slightly change the original conformation of Dbh. The flexible linker was predicted to have unconstrained flexibility (due to the small, non-polar amino acid, Gly) and favorable water interactions (due to Ser)^28^. The designed construct was assembled, expressed, and purified (Fig. 1B). A native Dbh was also expressed for comparison.

### Sso7d fusion increased the processivity of Dbh

**Figure 2:**
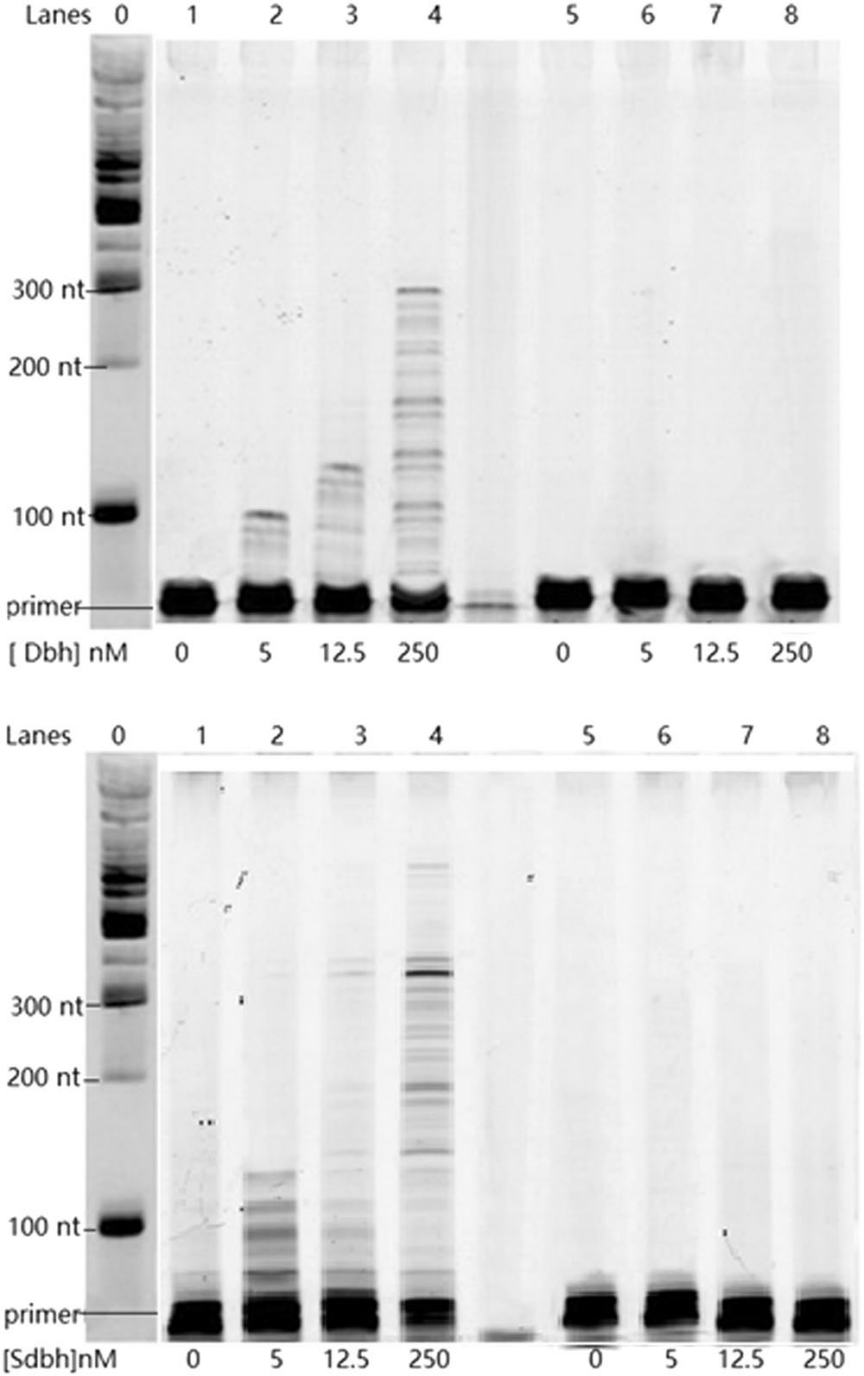
Processivity of Dbh and Sdbh. The ability of Dbh and Sdbh to extend a FAM-labeled primer (FAM-36 primer) annealed to single-stranded M13mp18 DNA from a single DNA binding event in the presence of a DNA trap. In lanes 1–4, the pol was preincubated with the primer-template DNA (12.5 nM) and the reactions were initiated by the addition of dNTPs (200 μM) and excess sperm DNA trap. As a control (lanes 5–8), the pol was preincubated with the primer-template DNA and the herring sperm DNA trap, and reactions were initiated by the addition of dNTPs. The intensity along the length of the lane was quantified and then the marker standards were used to assign a basepair length through the lanes using ImageQuant software. From there the greatest abundant product could be measured as the processivity. The unlabeled marker standards were visualized by SYBR stain independently and showed the length (nt) on the left of the first lane. (Note: the synthesized primer was 36 nt, but the FAM label altered its electrophoretic mobility, such that it ran at about 80 nt). See Supplemental S1 for full-length image.

Processivity was evaluated with the average extension length of products in the presence of excess herring sperm DNA as a trap which limited the pol from rebinding the DNA. Single-stranded M13mp18 was used as the template to estimate the processivity of native Dbh and fusion Sdbh (Fig. 2). The concentration of primer-annealed template (P-T) was fixed at 12.5 nM, and the amount of enzyme varied from 0 to 250 nM. When the pol was limiting (5 nM) and only one extension per DNA strand was expected, only a small fraction of FAM-labeled primers were extended, and the processivity of Dbh was estimated to be about 10 nt (the length of apparent FAM-labeled primer was 80 nt because the FAM fluorophore alters the electrophoretic properties). As expected, when the pol was not limiting (250 nM enzyme, 20-fold enzyme to P-T), Dbh synthesized replication products of several hundred bases in length, while at an equimolar pol-to-P-T ratio (12.5 nM enzyme), the processivity was about 30 nucleotides (Fig. 2), implying DNA molecules were extended multiple times.

Under the same assay conditions, when the Sso7d-fused pol was limiting (5 nM) and single extension events were favored, processivity of Sdbh increased to approximately 40 nt compared to 10 nt for Dbh (Fig. 2). When Sdbh was in a large molar excess and allowed multiple extension events per DNA strand (20-fold enzyme to P-T) the longest products were comparable to Dbh, but the 200 to 300 nt products were more intense. When the Sdbh was 12.5 nM, the processivity of Sdbh were approximately 2 fold higher than Dbh. Based upon the limiting pol conditions, we estimate that Sdbh incorporates 4-fold more nucleotides per extension than WTDbh. This is consistent with the idea of Sso7d as a processivity enhancer that helps maintain the LF domain and thumb domain around the DNA near the extending 3′ terminus (Fig. 1A).

### Identifying mutations in Dbh that increase DNA binding strength computationally

**Figure 3:**
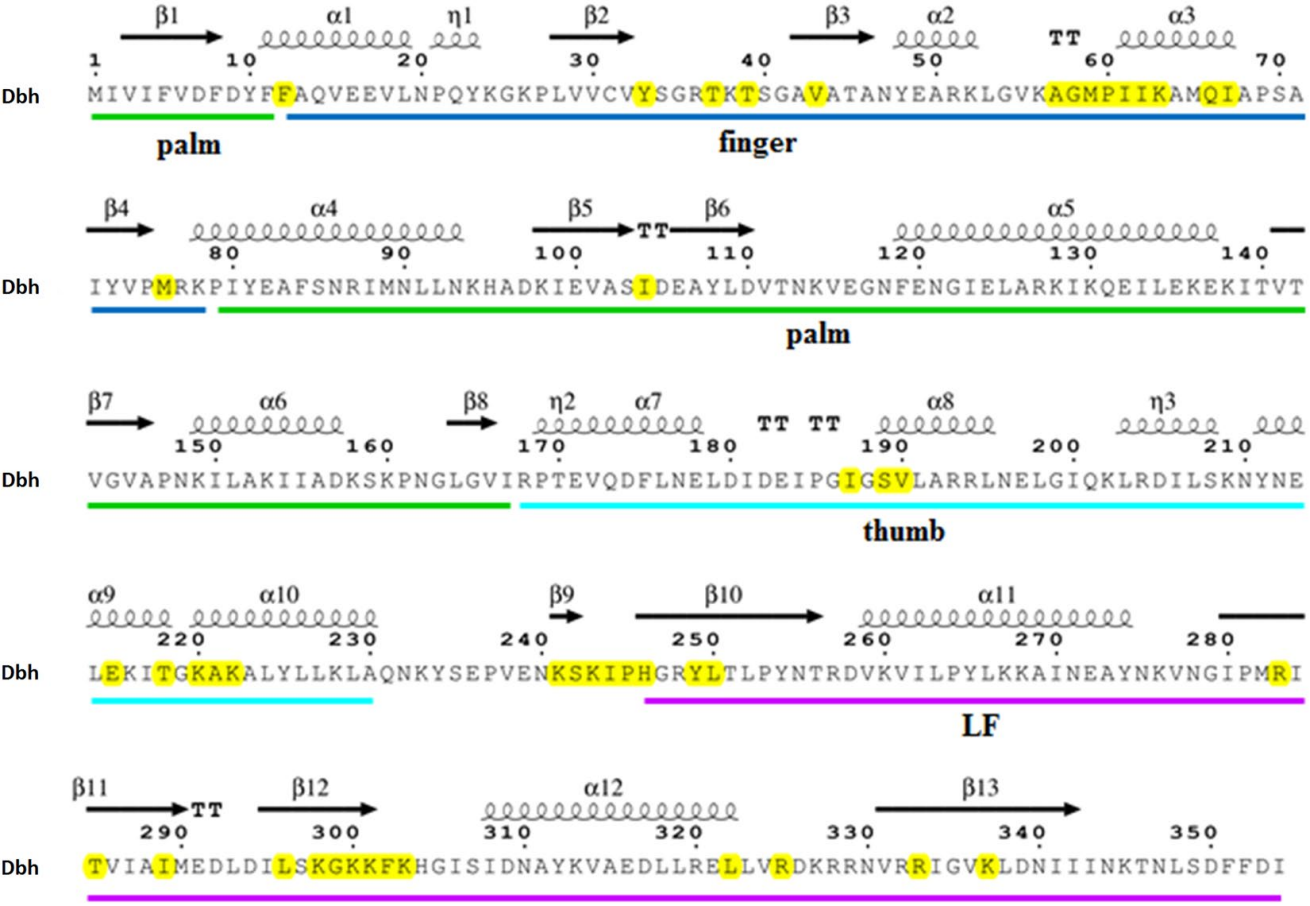
Non-conserved amino acids likely to interact with the DNA substrate. The Dbh amino acid sequence is shown with the domains indicated by color: palm (green), finger (blue), thumb (cyan), and LF (magenta). Non-conserved amino acids key to the interaction of Dbh and DNA duplex are highlighted in yellow. The secondary structures are indicated as coils (α-helices) and arrows (β-sheets) above the aligned primary amino acid sequence.

In order to determine the residues of Dbh that were most amenable to mutations, a multiple sequence alignment of known Y-family pols was used to identify conserved and non-conserved positions. 34 homologous sequences from Archaea, bacteria and eukaryotes with greater than 40% identity were generated. Since the residues located within 6 Å of the DNA duplex are generally considered important for the pol/DNA interaction^29^, the Dbh-DNA complex was analyzed to find the residues located within 6 Å of the double-stranded DNA. As shown in Fig. 3, 72 non-conserved residues in Dbh were found to be potential DNA contacts. Moreover, a library of potential mutation sites with mutation frequency (Table 1) was established based on the multiple sequence alignment and statistical analysis. The potential mutation sites were spread among the finger domain, thumb domain, the β-sheet of the LF domain, and the linker region of the thumb and LF domain. In addition, the contiguous residues K241, S242, K243, I244 and P245 had a high frequency mutation to RVRKS in several species (Table 1, R/24, V/19, R/25, K/25, S/18). These residues are in an interdomain linker that is a key determinant of pol conformation^30^. For each residue, a mutation to the highest frequency amino acid in the multiple sequence alignment was chosen. The mutations T37F, I62V, M76I, A221S, Y249I, L250V, K337R and KSKIP(241–245)RVRKS were made computationally, and alterations to the protein structure were calculated using Modeller 9.11.

**Table 1:** The mutant residue types and frequencies of the non-conserved amino acids (AA).

Position sites	Original	Mutant	Position sites	Original	Mutant
	AA	AA/frequency^(a)^		AA	AA/frequency^(a)^
12	F	Y/23, A/1	222	K	R/2
33	Y	F/18	241	K	R/24
37	T	F/16, N/3, P2, R1, S/1, E/1	242	S	V/19, T/1, Q/1, I/2, E/5, K/1
39	T	D/20, T/8, P7, K/2, N/4, R/2, S2	243	K	R/25, I/1, V/2
43	V	I/5	244	I	K/25, V/2, T/1, S/1, H/1
57	A	S/19	245	P	S/18, H/6, N/3
58	G	A/11	246	H	I/19, R/2, Q/1, M/1, F/2, Y/1
59	M	I/14	249	Y	I/17
60	P	A/1, S7	250	L	V/20
61	I	A8, C2, S1, L/3	283	R	A/14, K/1, S/1, T/4, E/4
62	I	V/14, P/6, S/2, Y/1, K/1	285	T	H/14, Y/5, S/3, A/3
63	K	R/3, E/11, Q/2	289	I	V/15
66	Q	E/12, K/13, N/1	296	L	V/26, I/2
67	I	L/3	298	K	R/23
76	M	I/7	299	G	S/5, E/3
104	I	V/1	300	K	R/15, Y/2, I/1
187	I	V/12	301	K	T/23, S/4
189	S	N/10, K/5, D/7, E/2, N/10, T/1	302	F	Y/2
190	V	I/15, M/1, S/4	303	K	P/12, T/6, N/2, G/3
215	E	K/20, V/2, S/1, A/1, I/2	322	L	I/25
218	T	I/20, V/5, L/3	325	R	E/19, K/3, S/2, A/1
220	K	R/2, E/16	333	R	E/1, A/1
221	A	S/9, K/1	337	K	R/28

^(a)^The mutant residue types and frequencies were statistically analyzed using HotSpot Wizard 1.7 software based on the results of the multiple sequence alignment.

Free binding energies of DNA to Dbh or the predicted mutants were estimated using the MM-PBSA method as described in Material and Methods. The predictions are listed in Table 2. All mutations except K337R and Y249I showed more favorable binding to the DNA template strand. With reference to native Dbh, the binding energy of the mutant KSKIP(241–245)RVRKS strengthened by - 532.2 kcal/mol, while for mutants K337R and Y249I, the binding energy weakened by 42.4 and 293.6 kcal/mol, respectively.

**Table 2:** Binding free energies and equilibrium dissociation constant of Dbh and the mutants to primer/template DNA.

Mutation sites	Primer/Template DNA^(a)^	Free binding energy Δ G_bind_ (kcal/mol)	(ΔG_bind_)^mutant^ − (ΔG_bind_)^wt^ (kcal/mol)	Variants	Equilibrium dissociation constant K_d_(nM)
WTDbh	P	−2251.8	0	WTDbh	54.8 ± 3.8
	T	−3465.7		Sdbh	51.2 ± 2.4
KSKIP(241–245) RVRKS	P	−2579.5	−532.2	SdbhKSKIP(241–245) RVRKS	34.8 ± 1.2
	T	−3670.2			
M76I	P	−2414.7	−380	SdbhM76I	42.8 ± 3.1
	T	−3682.8			
L250V	P	−2422.1	−318.3	SdbhL250V	50.4 ± 3.4
	T	−3603.7			
T37F	P	−2313.6	−159	SdbhT37F	49.7 ± 3.1
	T	−3562.9			
A221S	P	−2344.03	−100.45	SdbhA221S	47.5 ± 3.0
	T	−3473.91			
I62V	P	−2145.6	−60.3	SdbhI62V	49.8 ± 2.6
	T	−3632.2			
K337R	P	−2554.6	42.4	SdbhK337R	55.5 ± 4.1
	T	−3120.5			
Y249I	P	−2305.0	293.6	SdbhY249I	56.2 ± 3.4
	T	−3118.9			

^(a)^dsDNA (Primer/Template DNA) referred to the chain primer extended (P(5-3′)) and the corresponding template chain (T(3′-5′)).

### Experimental determination of the equilibrium dissociation constant (K_d_)

**Figure 4:**
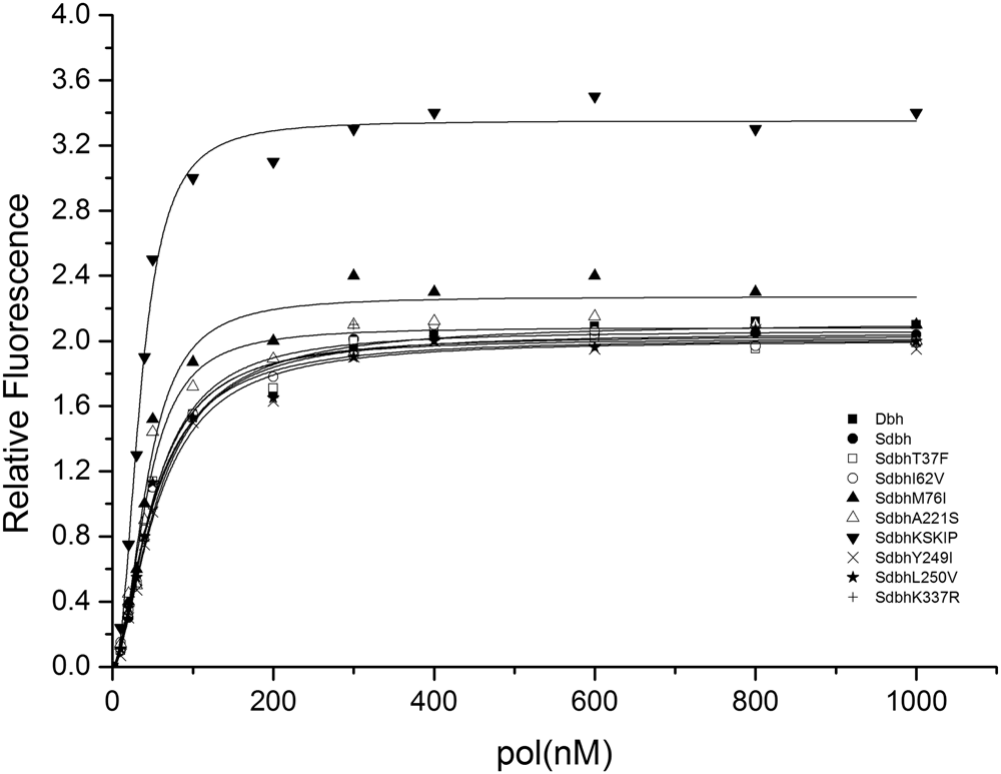
Equilibrium titrations of DNA substrate with Dbh and the variants. A constant amount of 2AP-P/T (100 nM) was titrated with increasing concentration of pol. The fluorescence was excited at 315 nm and observed at 370 nm. Each measurement was repeated four times, and the average value of the fluorescence intensity was recorded. A control experiment was performed with the non-fluorescent promoter DNAs under identical conditions. The fluorescence changes from the control experiments were subtracted from the data obtained with the 2AP-P/T, and the corrected values are plotted against [pol]. The analysis of the data yielded the dissociation constant K_d_ for Dbh(◾), Sdbh(⚫), SdbhT37F(◽) SdbhI62V(○) SdbhM76I(▴), SdbhA221S(▵), SdbhKSKIP241–245RVRKS(▾), SdbhY249I (×) and SdbhL250V (★), respectively.

To validate the computational prediction of the binding affinity of Dbh and the variants to template DNA, eight Sdbh variants (SdbhT37F, SdbhI62V, SdbhM76I, SdbhA221S, SdbhKSKIP(241–245)RVRKS, SdbhY249I, SdbhL250V, and SdbhK337R) were constructed, expressed and purified, and equilibrium titrations with a 2AP-P/T were performed, using 2AP attached to the primer as the probe. Excitation of 2AP-P/T at 315 nm can minimize protein absorption. The final equilibrium constant was obtained by subtracting the fluorescence of protein. Figure 4 shows a typical titration curve of the 2AP-P/T molecule with Dbh and the variants. The binding isotherms were fit to a hyperbola to calculate K_d_ values. As shown in Table 2, the measured K_d_ values for Dbh, Sdbh, SdbhM76I and SdbhA221S were 54.8 ± 3.8, 51.2 ± 2.4, 42.8 ± 3.1 and 42.5 ± 3.0 nM, respectively. The binding of Sdbh, SdbhM76I and SdbhA221S to the 2AP-P/T was a little stronger than that of WTDbh. However, the K_d_ value for SdbhKSKIP241–245RVRKS was 34.8 ± 1.2 nM, which indicated a significant increase in the affinity. This result is in agreement with that of the above binding energy calculation. Conversely, SdbhT37F, SdbhI62V and SdbhL250V showed almost identical affinity to that of Sdbh, while SdbhK337R and SdbhY249I showed less affinity than that of Sdbh. Thus variants SdbhKSKIP241–245RVRKS and SdbhM76I were subjected to further processivity analysis.

### Dbh variants with higher affinity can improve processivity

**Figure 5:**
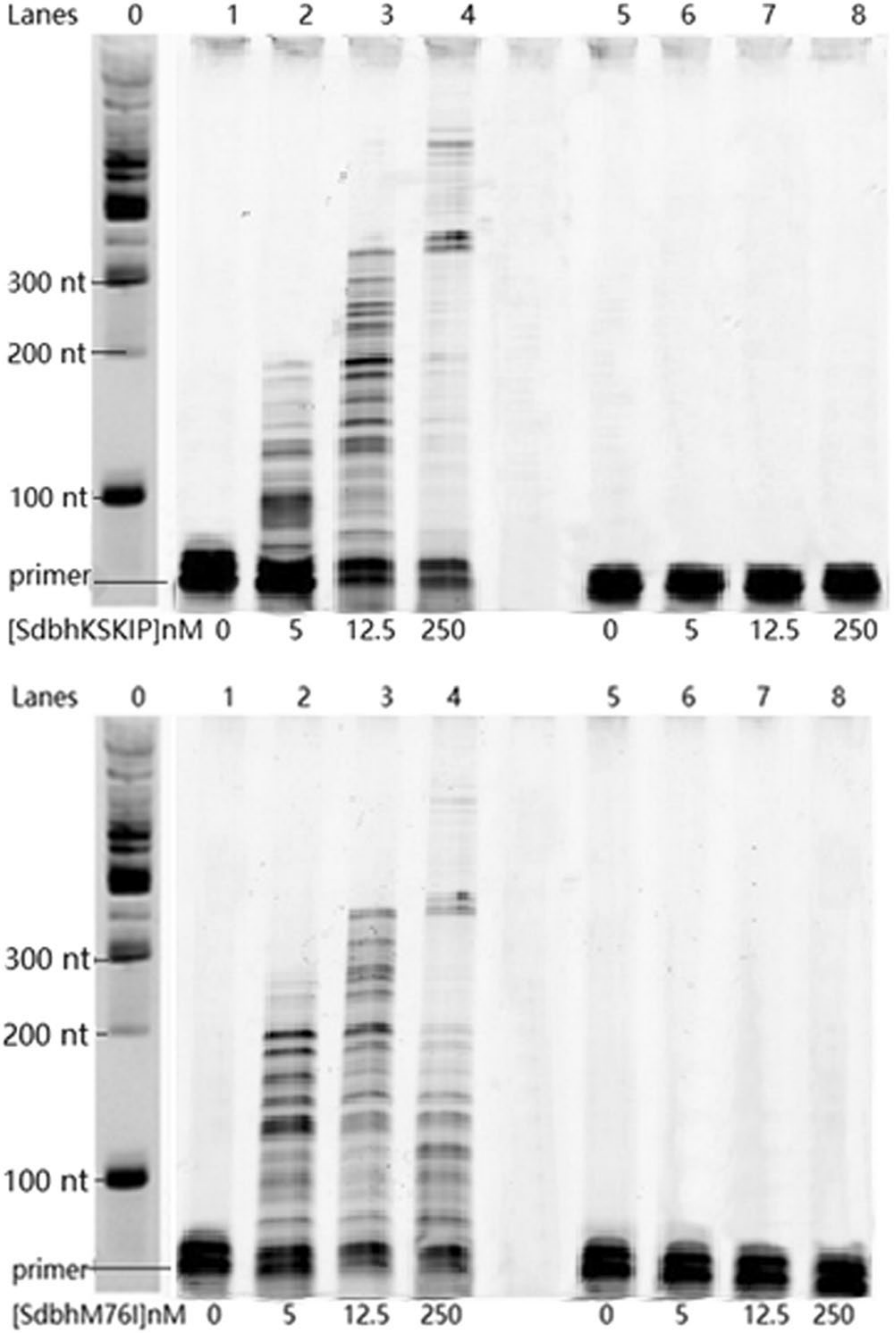
Processivity of Sdbh variants. The ability of the variants SdbhKSKIP241–245RVRKS and SdbhM76I to extend a FAM-labeled primer annealed to single-stranded M13mp18 DNA from a single DNA binding event in the presence of a DNA trap. The analysis method employed was the same as that used in Fig. 2. See Supplemental S1 for full-length image.

The processivity of the variants SdbhKSKIP241–245RVRKS and SdbhM76I were investigated using the same methods previously applied to Dbh and Sdbh. As shown in Fig. 5, when the pol was limiting, favoring single extensions (5 nM), the processivity of SdbhKSKIP(241–245)RVRKS and SdbhM76I were 60 and 100 nt, respectively, compared to 40 nt for Sdbh and 10 nt for Dbh. When the enzymes were 12.5 nM, SdbhKSKIP(241–245)RVRKS and SdbhM76I showed a similar processivity of about 140 nt, higher than that of Sdbh (60 nt). These results show that mutating KSKIP(241–245) and M76 can increase processivity beyond the improvements made by Sso7d alone. These results also demonstrate that increasing DNA binding strength can improve the processivity of Dbh.

### Steady-state kinetic analysis of one-base insertion

Velocity was studied as the activity of Dbh and the variants on the incorporation of dCTP (correct) and dATP (incorrect) opposite the G (normal) or 8-oxoG (damaged) template base. The polymerase assays and Michaelis–Menten plots were shown in Figure S2, from which V_max_ and K_m_ values were determined (Table 3). Data in Table 3 showed that the V_max_ for correct dCTP incorporation opposite the normal G base by SdbhKSKIP241–245RVRKS was higher than that of WTDbh (~2.3-fold), while the V_max_ for correct dCTP incorporation opposite the damaged 8-oxoG base was 2-fold higher than that of WTDbh. The V_max_ for incorrect dATP incorporation opposite the normal G base by SdbhKSKIP241–245RVRKS was ~8.4-fold higher than that of WTDbh, and dATP incorporation opposite the damaged 8-oxoG base was also higher (2.8-fold). The K_m_ values for correct dCTP incorporation and incorrect dATP incorporation opposite the normal G/damaged 8-oxoG base by SdbhKSKIP241–245RVRKS were all lower than that of WTDbh. Interestingly, the dATP misinsertion opposite 8-oxoG was more rapid than that opposite G by not only WTDbh but also Sdbh, SdbhM76I and SdbhKSKIP241–245RVRKS. These results indicated that increasing affinity can improve the activity of Dbh.

**Table 3:** Steady-state kinetics of incorporation of dCTP and dATP opposite G and 8-oxoG ﻿bases by Dbh and the variants

pol	Template base	dNTP	K_m_ (μM)^(a)^	V_max_ (nMmin^−1^)^(a)^	V_max_/K_m_ (min)
Dbh	G	dCTP	88.2 ± 12.0	1.0 ± 0.1	11
		dATP	215.2 ± 18.7	0.019 ± 0.001	0.088
	8-oxoG	dCTP	67.7 ± 6.3	0.5 ± 0.02	7.4
		dATP	139.8 ± 3.1	0.18 ± 0.002	1.3
Sdbh	G	dCTP	75.2 ± 12.0	1.4 ± 0.1	19
		dATP	128.4 ± 26.3	0.04 ± 0.003	0.31
	8-oxoG	dCTP	59.6 ± 11.1	0.61 ± 0.07	10
		dATP	114.9 ± 27.8	0.23 ± 0.009	2.0
SdbhM76I	G	dCTP	54.9 ± 8.6	1.9 ± 0.2	35
		dATP	76.7 ± 7.1	0.08 ± 0.01	0.10
	8-oxoG	dCTP	55.5 ± 4.9	1.0 ± 0.05	18
		dATP	69.1 ± 9.3	0.4 ± 0.05	5.8
SdbhKSKIP 241–245RVRKS	G	dCTP	40.1 ± 5.2	2.3 ± 0.1	57
		dATP	71.7 ± 9.6	0.16 ± 0.02	0.22
	8-oxoG	dCTP	33.5 ± 2.7	1.0 ± 0.04	30
		dATP	55.1 ± 2.9	0.5 ± 0.04	9.1

^(﻿a)^﻿The values of V_max_ and K_*m*_ were fit by nonlinear regression of the Michaelis-Menten equation. All reactions were done in duplicate, and the indicated data points are shown as the mean ± standard deviation.

## Discussion

Dbh is an *archaeal* representative of Y-family pols and exhibits a unique structure and set of properties (e.g. low processivity and fidelity)^13, 17^. Compared with classical pols, the Dbh active site is wider with fewer contacts to the DNA template and nucleotide substrates, which is useful for incorporating non-natural nucleotides. Our work was based on two observations: pols often require accessory proteins such as proliferating cell nuclear antigen (PCNA) to modify processivity^21^, and the residues present at the non-conserved positions of processive pols like *E*. *coli* Pol IV result in tighter binding and enhanced processivity^31^. Building on these observations, we show that enhancing interaction by fusing the processivity enhancer Sso7d to Dbh polymerase increases the nonspecific interaction with dsDNA, improving the processivity of the native Dbh. Further, creating bioinformatics-based mutations in non-conserved residues increased the DNA template binding ability and processivity of the Sso7d-Dbh fusion. We believe rational modifications and computational predictions such as these could allow for precise control over a range of DNA polymerase properties.

To understand the underlying elements that affect pols binding to their substrates, a large number of structures of pol-DNA complexes have been determined^32, 33^. These studies have shown that the pols share special substructures to recognize DNA, including β-sheets, α-helices, and loops. Forces like hydrophobic, van der Waals, and ionic interactions are attributed with stabilizing the pol-DNA complexes. Hydrogen bonding interactions between the pol and DNA bases in the major groove can mediate the binding specificity of the pol. When the MM-PBSA method was used to calculate the binding energy of the pol-DNA complex in this study, the intramolecular electrostatic and van der Waals, electrostatic and nonpolar contributions in liquid phase were the main concern. Other factors, e.g. non-electrostatic and non-specific components, that also affect DNA binding were ignored. This probably led to an inconsistency between the theoretical and the experimental data, although the calculated affinities in the present study are in agreement with the experimental K_d_. The mutant KSKIP(241–245)RVRKS showed the highest free energy (−532 kcal/mol) computationally, while the variant SdbhKSKIP(241–245)RVRKS showed the highest affinity (34.8 ± 1.2 nM) experimentally (Table 2).

However, the processivity of SdbhKSKIP(241–245)RVRKS was not the highest, which indicated the affinity is not the only factor that determines the processivity. Processivity of the DNA pols relates to the number of nucleotides added to the nascent strand during one round of binding and dissociation from the primer template. It is essential for processivity that the pols are binding to and sliding along the DNA. Tight binding of a pol to its DNA template is achieved through a large interaction surface, e.g. sequence-specific complexes which display complementarity in shape and polarity. For sliding, however, an enzyme must strike the right energetic balance so as to remain associated with its polymeric substrate, while retaining the ability to move from site to site. SdbhKSKIP(241–245)RVRKS exhibited high affinity, which probably hindered the pol from sliding on the template, and thus decreased the processivity.

Previous steady-state kinetics with Dpo4 showed 90-fold higher incorporation efficiency of dCTP over dATP opposite 8-oxoG, and also faster rates of dCTP incorporation opposite 8-oxoG compared to G^34^. In comparison to Dpo4, Dbh in this study also showed higher reaction velocities during dCTP incorporation vs. dATP opposite both G and 8-oxoG, and so did variants Sdbh, SdbhM76I and SdbhKSKIP 241–245RVRKS (Table 3). Moreover, the variant SdbhKSKIP 241–245RVRKS with the highest binding activity to DNA among Dbh, Sdbh, SdbhM76I and SdbhKSKIP 241–245RVRKS, showed the highest V_max_/K_m_ value both on dCTP incorporation opposite G/8-oxoG and on dATP incorporation opposite G/8-oxoG. These results further demonstrated that increasing binding strength can improve the activity of Dbh. However, the correlation between processivity and activity will be considered in greater detail elsewhere.

As applications requiring DNA polymerases to perform non-natural tasks increase, there will be a need for a molecular toolbox to tailor polymerase properties for different engineering applications. The current study shows two strategies for modifying DNA polymerase processivity. The use of protein fusions in tandem with computationally predicted mutations that improved binding affinity enhanced both processivity and catalytic efficiency in some cases. The approaches used here are likely generalizable to many different pols to increase processivity.

## Materials and Methods

### Construction of the Sso7d-dbh fusion (Sdbh)

The Dbh gene sequence (https://polbase.neb.com/polymerases/140-dbh) was synthesized by Integrated DNA Technologies Inc. (IDT) with a C-terminal 6× histidine tag. Previous work has shown that this tag does not significantly alter the polymerization activity of Y-family pols^35^. The *Sso7d* gene was synthesized by IDT based on the published amino acid sequence^24, 36^. Gibson assembly was employed to fuse Sso7d to the N-terminus of Dbh by a flexible linker (SLD, Table 4)^37^. The overlapping fragments of Dbh and Sso7d were PCR-amplified by oligonucleotides Fdbh-linker, Rdbh-dhfr, Fsso-dhfr and Rsso-linker, respectively (Table 4). The high copy plasmid DHFR (dihydrofolatereductase) supplied with PURExpress *In Vitro* Protein Synthesis Kit (New England Biolabs, Inc. (NEB)) was linearized by digestion with *Nde*I and *BamH*I restriction enzymes. The recombinant plasmids DHFR-dbh and DHFR-Sdbh were consequently constructed according to the Gibson assembly protocol (NEB) and verified by DNA sequencing.

**Table 4:** Oligonucleotides used in this study.

Oligo name	Oligo sequence (5′-3′)
Fdbh	GGAATTCCATATGCATCACCATCACCATCACATGATAGTGATATTCGTTGATT
Rdbh	CGCGGATCCTTAAATGTCGAAGAAATCAGAT
SLD	TCATCCGGTGGGGGAGGCTCTGGTGGTGGTGGTTCTGGTGGTGGTGGTTCTGGGATG
Fdbh-linker	TCATCCGGTGGGGGAGGCTCTGGTGGTGGTGGTTCTGGTGGTGGTGGTTCTGGGATGATGATAGTGATATTCGTTGA
Rdbh-dhfr	TAAAGGCCTCCTGCAGGTTAACCTTACTCGAGAATTCCCGGGATCC
	GTGATGGTGATGGTGATGTTAAATGTCGAAGAAATCAGAT
Fsso-dhfr	GTCTAGAAATAATTTTGTTTAACTTTAAGAAGGAGATATACATATGGCAACCGTAAAGTTCAAGTA
Rsso-linker	CATCCCAGAACCACCACCACCAGAACCACCACCACCAGAGCCTCCCCCACCGGATGACTTTTTCTGCTTCTCCAGC
FAM-36-primer	FAM-ACG CCT GTA GCA TTC CAC AGA CAA CCC TCA TAG TTA

### Protein expression and purification

The complete plasmids DHFR-dbh and DHFR-Sdbh were then transformed into *E*. *coli* strain BL21 (λDE3) (Invitrogen) for induced expression as described previously^13^. WT Dbh and fused Sdbh contained a 6× histidine tag on the N-terminus and were purified to electrophoretic homogeneity using nickel-nitrilotriacetic acid (Ni-NTA) permeation and Mono-S chromatography. Purified Dbh and Sdbh were stored in small aliquots at −80 °C in 50 mM Tris-HCl buffer (pH 7.7 at 22 °C) containing 50 mM NaCl, 1 mM dithiothreitol, and 50% glycerol (v/v).

### Mutation site identification and 3D-model determination

Using the Dbh amino acid sequence as a template, all DNA pols with over 40% homology were collected by BLAST from the NCBI database. A multiple sequence alignment was generated using Position Specific Iterated Blast (PSI-BLAST) and conservative sequence analysis was carried out on the alignment. The Dpo4 crystal structure (PDB: 4NLG) which shared a 99% sequence similarity with Dbh was used as a template for the following modeling and computation. A list of potential mutation sites was generated based on residues that were non-conserved and located within 6 Å of the DNA strand. The mutant residue types and frequencies were statistically analyzed using HotSpot Wizard 1.7 software (http://loschmidt.chemi.muni.cz/hotspotwizard/ActionServlet?action=protein&type=enzyme) based on the results of the multiple sequence alignment^38^. The 3D structures of mutant enzymes were constructed using the homology modeling program, Modeller 9.11, and then aligned to the selected model structure complex using the PyMol align tool (http://www.pymol.org)^39^. The binary complex of the mutant enzyme with the substrate was generated and exported by PyMol.

### Molecular Dynamics (MD) simulation and binding energy calculation

The Molecular Dynamics (MD) simulations, including energy minimization, system equilibration and production protocols, were performed with the GROMACS 4.5.5 package as described previously^40, 41^. Each enzyme-substrate complex was placed in a cubic box after adding GROMOS 9643a1 position, and filled with atomistic TIP3P water. A two-step energy minimization process was performed after system equilibration. The Molecular Mechanics Poisson–Boltzmann surface area (MM-PBSA) method, which has been widely used to predict the binding affinities for a variety of macromolecular complexes and protein-ligand complexes^41–43^, was employed to calculate the binding energy of different pol-DNA template complexes (DNA template in PDB: 4NLG is 5′-GAAGCCGGCGGAA-3′). In this study, the free energy of each molecule is defined as follows: Δ*G*_*binding*_ = *G*_*complex*_ − (*G*_*protein*_ + *G*_*ligand*_). Here, *G*_*complex*_, *G*_*protein*_, and *G*_*ligand*_ are the free energy of the polymerase-DNA complex, the free energy of the polymerase, and the free energy of DNA, respectively. The free energy *G* can be calculated by the following scheme, based on the MM-PBSA method: G = *E*_*MM*_ *TS*_*MM*_ + *G*_*solv*_, where *E*_*MM*_ is comprised of the intramolecular electrostatic (*E*_*elec*_) and van der Waals (*E*_*vdW*_) interaction energies. The free energy of solvation, *G*_*solv*_, was approximated as the sum of electrostatic and nonpolar contributions in liquid phase. *TS*_*MM*_ was ignored as it did not contribute significantly to the binding energy in these conditions. All binding energies were calculated by Gromacs 4.5.5 combined with the *g_mmpbsa* program developed by Kumari *et al*.^44^.

### Site-directed mutagenesis of Sdbh

Guided by the computational predictions, the mutations with decreased binding energy were subjected to site-directed mutagenesis. Mutagenesis was performed using a QuikChange site-directed mutagenesis kit by Bio Basic Inc. (Ontario, Canada), and sequences were verified prior to bacterial expression. Mutant proteins were expressed, purified, and stored using the same procedure as for the WT enzyme. These Sdbh variants were then used to verify the residues in Dbh that were most critical for binding to the DNA template.

### Measuring binding of Dbh and the mutants

Fluorescence titrations were performed to determine the equilibrium dissociation constant (K_d_) of Dbh and the mutants as previously described^45, 46^. Primer-template DNA with 2-aminopurine (2AP) located at the primer terminus (2AP-P/T) was prepared by Sangon Biotech (Shanghai, China).This 2AP-P/T was excited at 315 nm and the emission was observed at 370 nm. Fluorimetric titration experiments were performed on a Perkin Elmer LS50B Luminescence Spectrometer. A constant amount of 2AP-P/T (100 nM) was titrated against increasing concentration of Dbh or the mutants (0–1000 nM) in the reaction buffer (50 mM Tris acetate, pH 7.5, 50 mM sodium acetate, 10 mM magnesium acetate, 5 mM dithiothreitol, 0.05% Tween 20) at 25 °C. A control experiment was carried out in identical conditions with the presence of the unlabeled P/T (100 nM) and increasing amounts of the pols. The fluorescence changes from the control experiments were subtracted from the data obtained with the 2AP-P/T, and the corrected values were plotted against the corresponding pol concentration. The dissociation constant K_d_ was calculated using the following equation, ${\rm{F}}=\frac{{F}_{max}\times [pol]}{{K}_{d}+[pol]}$, where F is the relative fluorescence intensity, and F_max_ is its maximum value.

### Primer and template DNA used in polymerase assays

All DNA substrates used in the extension assays are listed in Table 4 and were synthesized from IDT. The primers used for processivity assays and steady-state kinetic analyses were synthesized with a 5′-6-carboxyfluorescein (FAM) label for fluorescence visualization. Circular single-stranded M13mp18 DNA was used as the template in processivity assays and purchased from Bayou Biolab. Primer was annealed to the template at a 1:1.5 ratio in an annealing buffer containing 10 mM HEPES-NaOH (pH 7.4) and 50 mM NaCl. The annealed complex was heated to 95 °C for 5 min, cooled to 60 °C at 0.1 °C/s, incubated at 60 °C for 10 min, cooled again to 4 °C at 0.1 °C/s, and then stored at −20 °C until use. The annealed primer-template was thawed on ice immediately before assays.

### Processivity assay

The processivity assay was performed in the presence of a trap to prevent rebinding^19, 35, 47, 48^. The pol at concentrations from 5 nM to 250 nM was preincubated with the FAM-labeled primer-template(12.5 nM) in the reaction buffer (10 mM HEPES-NaOH (pH 7.4), 50 mM NaCl, 10 mM MgCl_2_, 1 mM DTT, 100 μg/mL BSA, and 0.1% Triton X-100). Reactions were initiated by adding 200 μM dNTPs and a 400-fold excess of herring sperm (Promega) as a trap. After incubation for 5 min at 37 °C, reactions were terminated by the addition of 10 μL of 95% formamide, 10 mM EDTA, and the reaction products were denatured at 100 °C for 5 min, then briefly chilled on ice. Reaction mixtures were separated via electrophoresis using 10% TBE-Urea precast polyacrylamide gels (Bio-Rad). The amounts of FAM fluorescence in the unextended and extended primer bands were quantitated using a Typhoon 9400 scanner and ImageQuant software (GE Healthcare). A distribution of DNA lengths were present, and processivity values were reported as approximately 85 percentile of the distribution.

### Steady-state kinetic analysis of one-base insertion

Steady-state kinetic parameters were analyzed for incorporation of dCTP and dATP opposite the undamaged G or damaged 8-oxoG (7,8-dihydro-8-oxyguanine) template base, and assays were performed using established methods^49–51^. Specifically, each reaction contained 1.0 μM of the annealed primer-template (the primer was 5′-FAM-GGTTGGATGGTAG-3′, the template was 5′-CTAACXCTACCATCCAACC-3′, X represents oxoG/G). Dbh and the variants (20 nM) were preincubated with the primer-template in the buffer (40 mM Tris-HCl buffer (pH 7.5), 100 mM KCl, 5 mM MgCl_2_, 10 mM DTT, 0.1% Triton X-100, 50 μg/μl BSA). Reactions were initiated by the addition of varying concentrations of a single dCTP or dATP, incubated for 5 min at 37 °C, and then quenched by the addition of 10 μL of 95% formamide, 10 mM EDTA. Substrate and product DNA were then separated by electrophoresis on a 20% polyacrylamide (w/v) containing 7 M urea gel. Fluorescence in the substrate and product primer bands was scanned using the Typhoon 9400 scanner (GE Healthcare) and quantified by ImageQuant software. The reaction rates (v, nM/min) were plotted as a function of the dNTP concentration, and the data were fit by nonlinear regression of the Michaelis-Menten equation, ${\rm{v}}=\frac{{V}_{max}\times [dNTP]}{{K}_{m}+[dNTP]}$, to calculate apparent K_m_ and V_max_ steady-state parameters.

## Electronic supplementary material

Supplemental Figure legends

Full image of Dbh

Full image of Sdbh

Full image of SdbhKSKIP

Full image of SdbhM76I

Steady-state kinetic analysis of dCTP and dATP incorporation on unmodified G and oxoG-modified templates by Dbh and the varitants.
