# Supplementary material for: DNA binding strength increases the processivity and activity of a Y-Family DNA polymerase
Source: Sci Rep. 2017 Jul 6;7:4756. doi: 10.1038/s41598-017-02578-3 (PMC5500549; doi:10.1038/s41598-017-02578-3)
Supplement: Supplementary file 1 — Supplemental Figure legends [file 41598_2017_2578_MOESM1_ESM.pdf]

**DNA binding strength increases the processivity and activity of a Y-Family DNA polymerase**

Jing Wu[1,2], Alexandra De Paz[1], Bradley M. Zamft[3], Adam H. Marblestone[4,5], Edward S. Boyden[6,7,8], Konrad P. Kording[9,10]and Keith E.J. Tyo*[1]

1 Department of Chemical and Biological Engineering, Northwestern University, Evanston, Illinois, United States of America,

2 The school of pharmacy, Jiangnan University, Wuxi, Jiangsu Province, China,

3Department of Genetics, Harvard Medical School, Boston, Massachusetts, United States of America,

4 Biophysics Program, Harvard University, Boston, Massachusetts, United States of America,

5Wyss Institute, Harvard University, Boston, Massachusetts, United States of America,

6 Media Lab, Massachusetts Institute of Technology, Cambridge, Massachusetts, United States of America,

7 Department of Biological Engineering, Massachusetts Institute of Technology, Cambridge, Massachusetts, United States of America,

8 McGovern Institute, Massachusetts Institute of Technology, Cambridge, Massachusetts, United States of America,

9 Department of Physiology, Northwestern University, Chicago, Illinois, United States of America,

10 Department of Applied Mathematics, Northwestern University, Chicago, Illinois, United States of America

Manuscript in preparation for:
Scientific Reports

*Corresponding author:
Telephone: 847.868.0319
Fax: 847.467.2972
email: k-tyo@northwestern.edu

**Supplemental Figure 1. Full-length images on processivity of Dbh and the variants.** (A)Dbh, (B) Sdbh,(C) SdbhKSKIP241-245RVRKS, (D) SdbhM76I.

**Supplemental Figure2. Steady-state kinetic analysis of dCTP and dATP incorporation on unmodified G and oxoG-modified templates by Dbh and the varitants.** The reaction contained a template with G or 8-oxoG at position X as follows 5'-FAM-GGTTGGATGGTAG-3' and 5'-CTAAC<u>X</u>CTACCATCCAACC-3'.The PAGE gels (top panels) show data as a function of dCTP/ dATP concentration, while the plots (bottom panels) measure the log of the frequency of nucleotide insertion for dCTP/ dATP. The labeled (-1) position represents the 13-mer primer with the 3'-end positioned one base before the G or oxoG; The labeled (0) position represents the 14-mer products with one base opposite G or oxoG; See Table 3 for $V_{max}$ and $K_m$ values.
