# Supplementary figures and images for: DNA binding strength increases the processivity and activity of a Y-Family DNA polymerase
Source: Sci Rep. 2017 Jul 6;7:4756. doi: 10.1038/s41598-017-02578-3 (PMC5500549; doi:10.1038/s41598-017-02578-3)

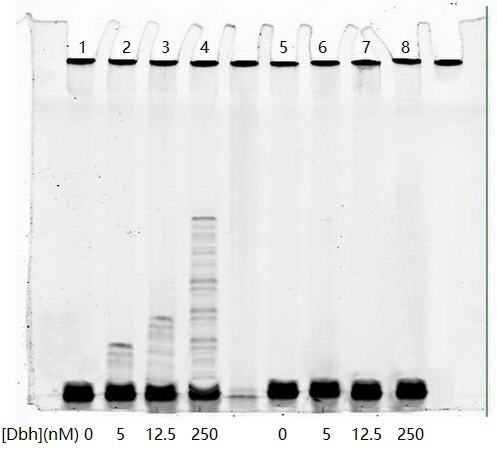

Supplement: Supplementary file 2 — Full image of Dbh [file 41598_2017_2578_MOESM2_ESM.tif]

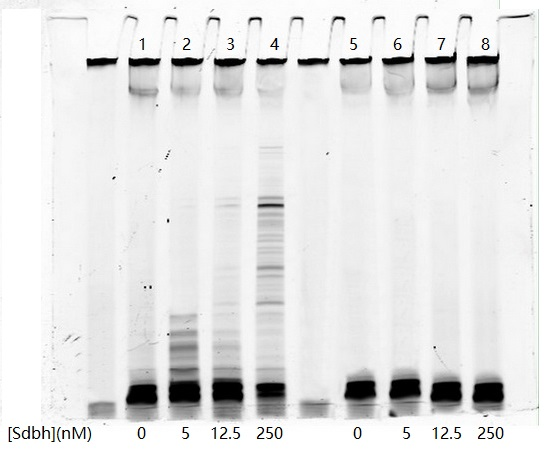

Supplement: Supplementary file 3 — Full image of Sdbh [file 41598_2017_2578_MOESM3_ESM.tif]

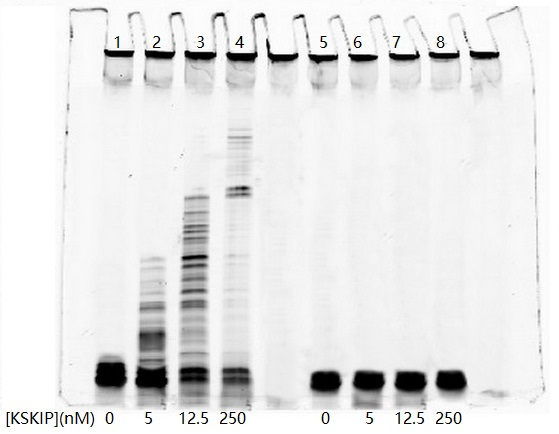

Supplement: Supplementary file 4 — Full image of SdbhKSKIP [file 41598_2017_2578_MOESM4_ESM.tif]

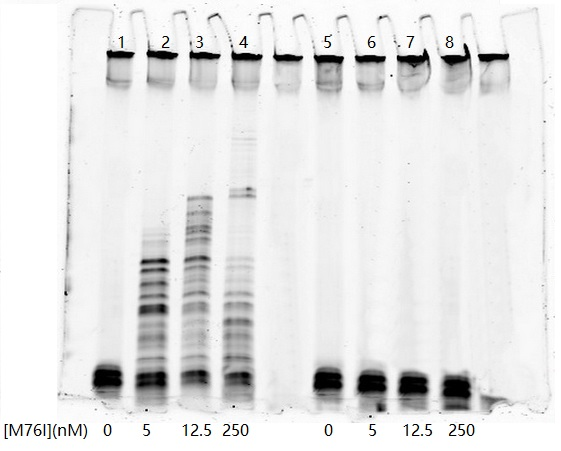

Supplement: Supplementary file 5 — Full image of SdbhM76I [file 41598_2017_2578_MOESM5_ESM.tif]

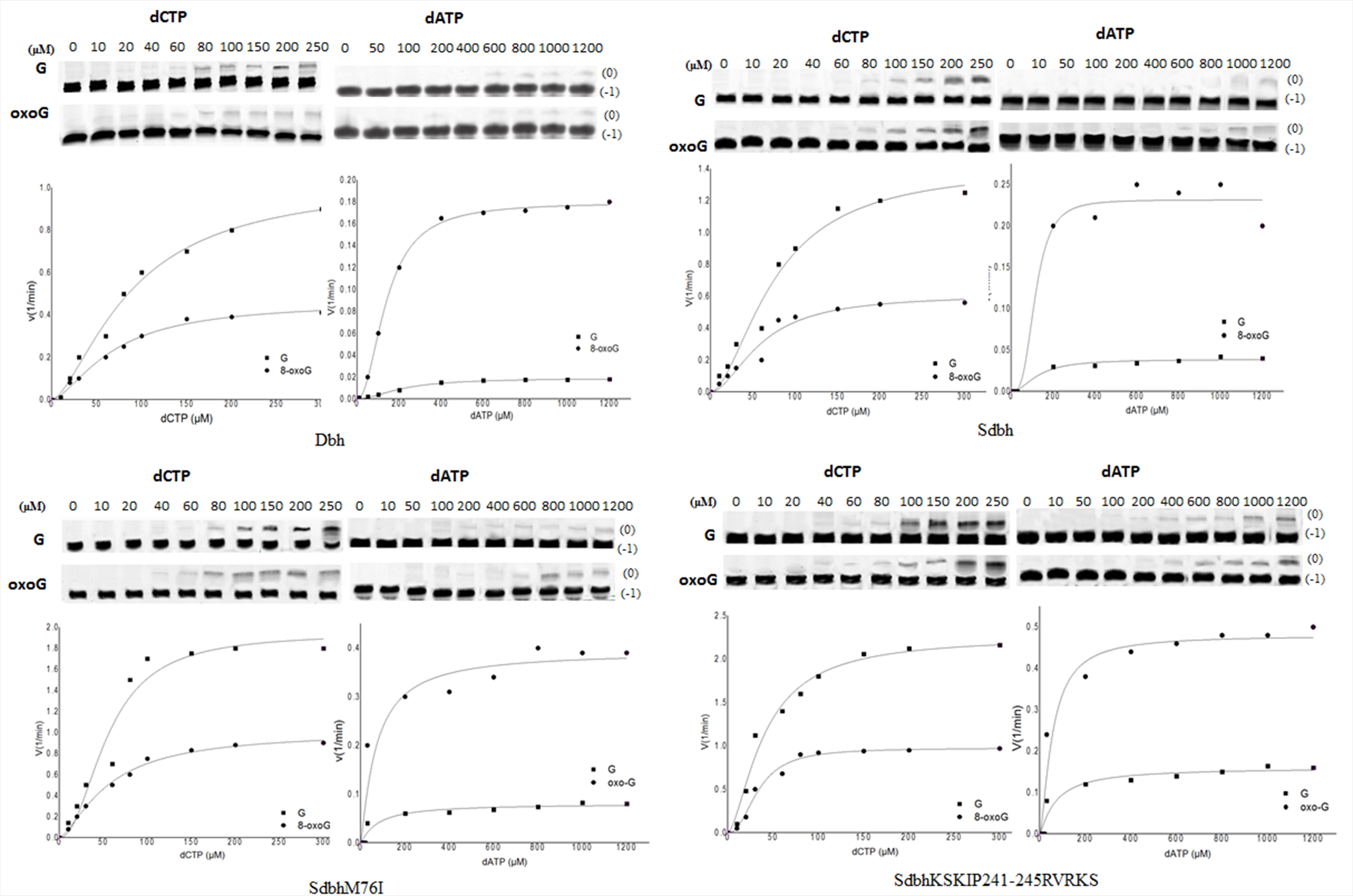

Supplement: Supplementary file 6 — Steady-state kinetic analysis of dCTP and dATP incorporation on unmodified G and oxoG-modified templates by Dbh and the varitants. [file 41598_2017_2578_MOESM6_ESM.tif]
